# Effects of Mo, Nb, Ta, Ti, and Zr on Mechanical Properties of Equiatomic Hf-Mo-Nb-Ta-Ti-Zr Alloys

**DOI:** 10.3390/e21010015

**Published:** 2018-12-25

**Authors:** Ko-Kai Tseng, Chien-Chang Juan, Shuen Tso, Hsuan-Chu Chen, Che-Wei Tsai, Jien-Wei Yeh

**Affiliations:** 1 Department of Materials Science and Engineering, National Tsing Hua University, Hsinchu 30013, Taiwan; 2 High Entropy Materials Center, National Tsing Hua University, Hsinchu 30013, Taiwan

**Keywords:** high-entropy alloys, refractory high-entropy alloys, alloys design, elevated-temperature yield strength, solid solution strengthening effect

## Abstract

Nowadays refractory high-entropy alloys (RHEAs) are regarded as great candidates for the replacement of superalloys at high temperature. To design a RHEA, one must understand the pros and cons of every refractory element. However, the elemental effect on mechanical properties remains unclear. In this study, the subtraction method was applied on equiatomic HfMoNbTaTiZr alloys to discover the role of each element, and, thus, HfMoNbTaTiZr, HfNbTaTiZr, HfMoTaTiZr, HfMoNbTiZr, HfMoNbTaZr, and HfMoNbTaTi were fabricated and analyzed. The microstructure and mechanical properties of each alloy at the as-cast state were examined. The solid solution phase formation rule and the solution strengthening effect are also discussed. Finally, the mechanism of how Mo, Nb, Ta, Ti, and Zr affect the HfMoNbTaTiZr alloys was established after comparing the properties of these alloys.

## 1. Introduction

Refractory elements, including Rhenium (Re), Molybdenum (Mo), Niobium (Nb), Tantalum (Ta), and Tungsten (W) [1] are very important for improving mechanical properties in advanced alloys such as Titanium alloys and Nickel-base superalloys. Generally, elements with a melting point higher than Titanium (Ti), such as Chromium (Cr), Hafnium (Hf), Osmium (Os), Ruthenium (Ru), Vanadium (V), and Zirconium (Zr) are also classified as refractory elements. In Titanium alloys, refractory elements, especially Mo and W, are all beta stabilizers and possess a strong solid solution strengthening characteristic [2]. Nb forms Ni_3_Nb *γ* phase in Nickel-base superalloys such as Inconel 718 [3]. The improved creep resistance of the sixth generation superalloy TMS-238 mainly results from Re and Ru additions [4]. Refractory alloys also play significant roles in industry. In the second generation nuclear power plants, the most-used materials for fuel cladding are Zircoloy 2 and Zircoloy 4. The Niobium alloy C103 is used for the nozzle extension of satellites [5]. Refractory alloys are also known as having the great potential for elevated-temperature applications because of their high strength at elevated temperature. According to thermodynamics, thermal efficiency of a turbine engine could be enhanced by increasing the turbine inlet temperature. However, the melting point of Nickel-base superalloy limits the application itself above 1200 °C. Therefore, it is necessary to develop new refractory alloys, especially for applications at temperatures higher than 1200 °C.

In 2004, Professor Yeh and his group published the concept of high-entropy alloys (HEAs) [6]. He defined high-entropy alloys (HEAs) as having five or more major elements beneath 5–35 at.%, and minor elements below five at.%. In 2006, he also established four core effects of HEAs [7]: High-entropy, severe-lattices-distortion, sluggish-diffusion, and the cocktail effect. With the new concepts, scientists are able to develop and expand alloys without restrictions [8,9,10,11,12]. Some HEAs have been found to have attractive properties on diffusion [13], oxidation [14], corrosion [15], fatigue [16], creep [17], fracture toughness [18], and elevated-temperature strength [19]. High-entropy superalloys (HESAs) [20], eutectic HEAs (EHEAs) [21], light-weight HEAs (LWHEAs) [22], refractory high-entropy alloys (RHEAs) [23], etc. have been proposed and attract increased attention. In 2010, Senkov and Miracle first published RHEAs, MoNbTaW and MoNbTaVW [23,24]. These two alloys possess body-centered cubic (BCC) structure and have excellent elevated-temperature yield strength which is around 400 MPa at 1600 °C. But their density is much higher, and the room temperature compressive ductility is very low. In 2012, they published the equiatomic composition of HfNbTaTiZr, which possesses excellent compressive ductility up to 50%, lower density but poor elevated-temperature yield strength [25,26]. From then on, there were over 150 papers published concerning RHEAs [19].

There are some researches working on the addition of Al [27,28], Mo [29], Ti [30], V [31], or Zr [32], but few of them focus on the elevated-temperature mechanical performance and the overall effect of the constituent elements. To understand the elemental effect on mechanical properties, equiatomic HfMoNbTaTiZr alloy is firstly designed as a base alloy by adding the high modulus refractory element Mo to HfNbTaTiZr. Then the subtraction method is used to analyze each elemental effect in the equiatomic HfMoNbTaTiZr. Thus, in this study, six alloys HfMoNbTaTiZr, HfNbTaTiZr, HfMoTaTiZr, HfMoNbTiZr, HfMoNbTaZr, and HfMoNbTaTi are investigated and compared on their microstructure. Further, the compressive properties at room temperature and at elevated temperature are investigated. By comparing HfMoNbTaTiZr and HfNbTaTiZr, the influence of the addition of Mo can be understood. Likewise, the influence of the addition of Nb, Ta, Ti, or Zr can be articulated. In addition, promising compositions were found and further improved design is suggested. The solid solution phase formation rule and the solid solution strengthening effect will be discussed.

## 2. Materials and Methods 

The experimental Hf-Mo-Nb-Ta-Ti-Zr alloy series was prepared by vacuum-arc melting. The purity of raw elements including Hf, Mo, Nb, Ta, and Zr was 99.9 wt.%, and that of Ti was 99.99 wt.%. The melting points of each constituent element used in alloys are shown in Table 1. These pure metals were stacked together in the sequence of low melting point to high melting point from bottom to top. The stacked metals were melted together in a water-cool copper mold and solidified therein. The ingot of each alloy was flipped and re-melted, at least, four times to improve the chemical homogeneity. The crystal structure of the alloy samples taken from the portion near the copper mold was examined with the Shimadzu XRD-6000 X-ray diffractometer (SHIMADZU CORPORATION, Kyoto, Japan), operated at 30 kV and 20 mA with a scanning rate of 4°/min from 20° to 100°. JEOL JSM-5410 (JEOL Ltd., Tokyo, Japan) scanning electron microscope (SEM) and JXA-8500F FE-EPMA (JEOL Ltd., Tokyo, Japan) was used to analyze the samples in backscattering electron (BSE) mode. Energy dispersive spectrometry (EDS) was also used to confirm the chemical compositions. The cylindrical samples for the compression test were 3.6 mm in diameter and 6 mm in height. The room temperature compression tests were conducted with Instron 4468 (INSTRON, Norwood, MA, USA) universal testing machine, and the high temperature compression tests were performed on Gleeble-3500 (DYNAMIC SYSTEMS INC, Poestenkill, NY, USA) thermal–mechanical simulator. All tests were examined at the crosshead speed of 0.36 mm/min, which imposed the strain rate of 10^−3^ s^−1^ on the samples.

## 3. Results

**Figure 1:**
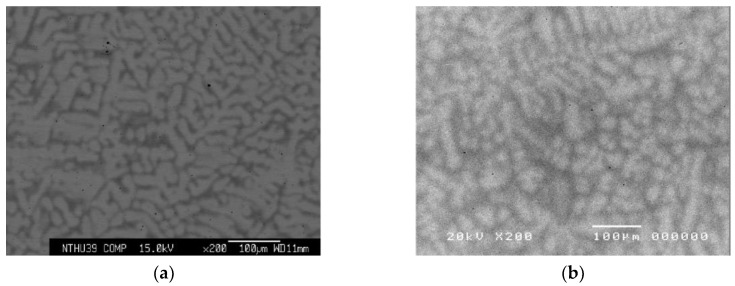

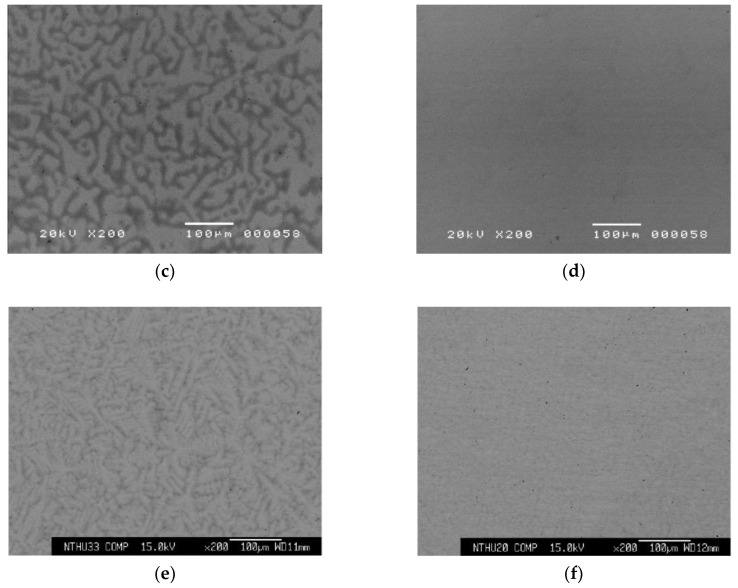
Backscattering electron (BSE) images of (**a**) HfMoNbTaTiZr, (**b**) HfNbTaTiZr, (**c**) HfMoTaTiZr, (**d**) HfMoNbTiZr, (**e**) HfMoNbTaZr, and (**f**) HfMoNbTaTi. All the alloys show the dendritic structure except HfMoNbTiZr.

Figure 1a–f are BSE images of experimental Hf-Mo-Nb-Ta-Ti-Zr alloys. A typical dendritic structure is observed. Their compositions as obtained from SEM-EDS are shown in Table 1. It is noted that the dendritic area is rich in Ta and Mo which have the highest two melting points. This is expected since high melting point elements tend to crystalize first during solidification. By contrast of dendritic structure is poor in HfMoNbTiZr alloy as observed in the BSE image. This means the partition between dendrite and interdendrite is small and the coring phenomenon was less obvious. This is due to the subtraction of the highest melting point element Ta which would solidify first with a Ta-rich solid solution.

**Figure 2:**
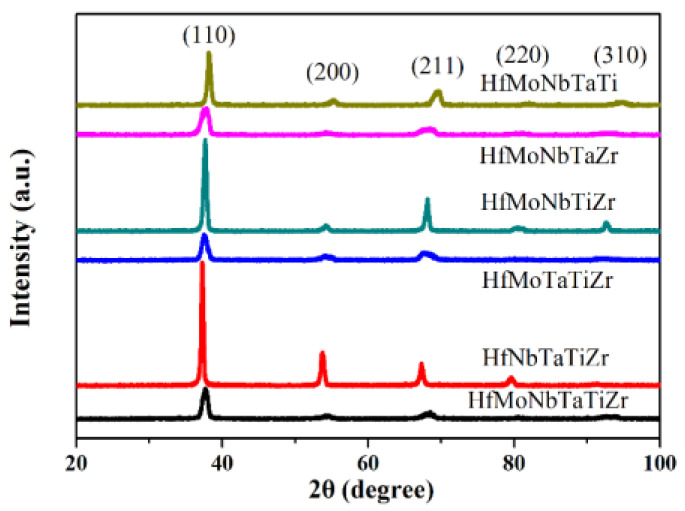
X-ray diffraction patterns of Hf-Mo-Nb-Ta-Ti-Zr alloy series.

Figure 2 shows the X-Ray diffraction patterns. The main phase of the Hf-Mo-Nb-Ta-Ti-Zr alloy series is a BCC disordered solid solution. The asymmetry of (200) and (211) peaks are shown in the diffraction pattern results from the cored dendritic structure. The composition variation of dendritic and interdendritic areas causes a little difference in the lattice constant. The lattice constants of the Hf-Mo-Nb-Ta-Ti-Zr alloy series listed in Table 2 are in the range of 3.305 to 3.400 Å calculated by the Nelson–Riley extrapolation function. Referring to the phase diagrams of each binary alloy between Hf, Mo, Nb, Ta, Ti, and Zr, Hf-Mo, and Mo-Zr binary alloys form Mo_2_Hf and Mo_2_Zr, respectively, in a certain range of composition even at high temperature. However, these two intermetallic compounds or others do not show up in Hf-Mo-Nb-Ta-Ti-Zr alloys, which means the high entropy effect has a significant benefit in forming simple BCC solid solution in this alloy system especially at high temperature.

**Figure 3:**
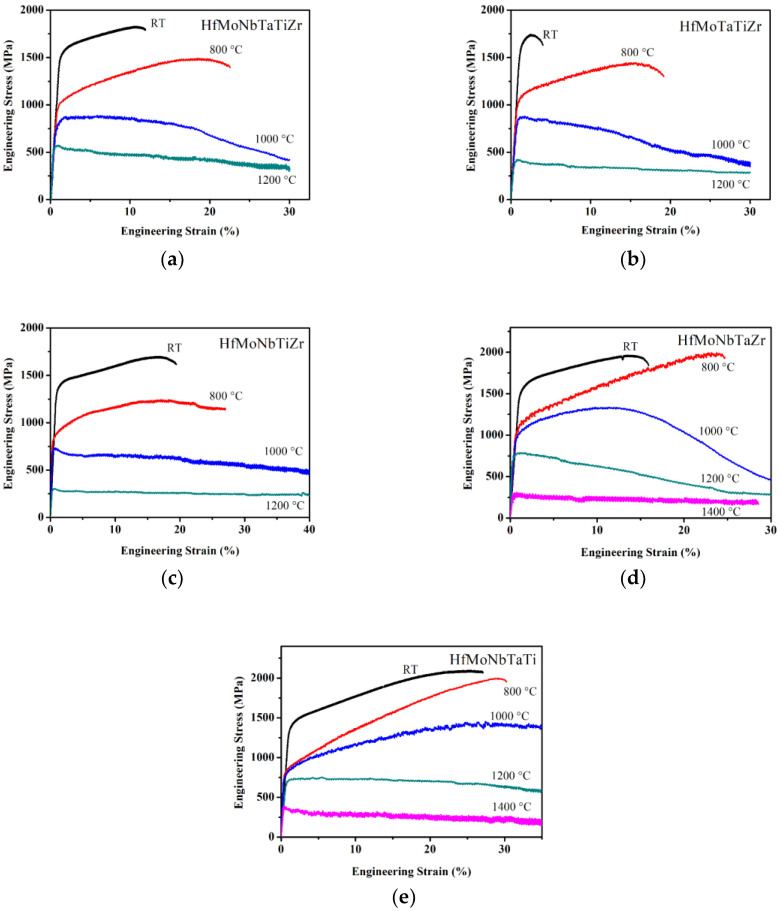
Engineer compressive stress–strain curve of (**a**) HfMoNbTaTiZr, (**b**) HfMoTaTiZr, (**c**) HfMoNbTiZr, (**d**) HfMoNbTaZr, and (**e**) HfMoNbTaTi.

Figure 3 shows the compression test results of Hf-Mo-Nb-Ta-Ti-Zr alloy series. At room temperature, the yield strength of HfMoNbTaTiZr alloy was 1512 MPa, and ultimate strength was 1828 MPa when the strain was 11%. The compression tests for HfMoNbTaTiZr alloy were also conducted at 800 °C, 1000 °C, and 1200 °C, respectively. At 800 °C, the yield strength of HfMoNbTaTiZr alloy was 1007 MPa and ultimate strength was 1489 MPa when the strain was 19%, which shows obvious work hardening. At 1000 °C and 1200 °C, the results of yield strength were 814 MPa and 556 MPa, respectively, but the strength kept decreasing from the yield point to the end of the test, showing the work softening behavior. No crack was observed at 1000 °C and 1200 °C.

When an element is removed from HfMoNbTaTiZr, the behavior is changed. The subtraction of Nb gives HfMoTaTiZr alloy. At room temperature, the yield strength of HfMoTaTiZr alloy was 1600 MPa, and the ultimate strength was 1743 MPa when the strain was 3%. At 800 °C, the yield strength was 1045 MPa, and the ultimate strength was 1446 MPa when the strain was 23%. As for the HfMoNbTaTiZr alloy, the stress–strain curve of HfMoTaTiZr alloy shows obvious work hardening effect. The results of yield strength were 855 MPa and 404 MPa at 1000 °C and 1200 °C, respectively. The strength kept decreasing from the yield point to the end of the test. No crack was observed at 1000 °C and 1200 °C.

The subtraction of Ta from HfMoNbTaTiZr gives HfMoNbTiZr. At room temperature, the yield strength of alloy was 1351 MPa, and the ultimate strength was 1698 MPa when the strain was 17%. This alloy performs with better toughness than HfMoNbTaTiZr and HfMoTaTiZr does at room temperature. At 800 °C, the yield strength was 829 MPa and the ultimate strength was 1244 MPa when the strain was 18%. At 1000 °C, yield strength was 721 MPa, and the strength kept decreasing when the strain increased. At 1200 °C, the yield strength was 301 MPa. The strength of the alloy was almost constant after the yield point to the end of the test. The strain softening effect was balanced by the strengthening effect.

Ti was removed in sequence. At room temperature, the yield strength of HfMoNbTaZr alloy was 1524 MPa, and the ultimate compress strength was 1963 MPa when the strain was 13.5%. At 800 °C, the compressive yield strength was 1005 MPa, and the ultimate strength was 1991 MPa when the strain was 24%. As with HfMoNbTaTiZr, there is an obvious work hardening effect shown at 800 °C in the stress–strain diagram. The yield strength was 927 MPa at 1000 °C. There was still a work hardening effect at the beginning of the test. The ultimate strength was 1336 MPa when the strain was 11%, but the strength decreased drastically to 464 MPa at the end of the test. At 1200 °C, the yield strength was 694 MPa, but the strength decreased to 289 MPa when the test stopped at 30% strain. At 1400 °C, the yield strength was 278 MPa, and the strength barely decreased during the test. Except for 800 °C, no fracture was observed during the test at elevated temperatures.

Finally, Zr was removed. At room temperature, the yield strength of HfMoNbTaTi alloy was 1369 MPa, and the ultimate compress strength was 2094 MPa when the strain was 25%. The yield strength at 800 °C was 822 MPa, and the ultimate strength was 1998 MPa when the strain was 29%. An obvious work hardening effect can be observed in the stress–strain curve. At 1000 °C, the yield strength was 778 MPa, and there was still work hardening effect at the beginning of the test until the ultimate strength 1454 MPa, at 27.5% strain. The results of yield strength were 699 MPa and 367 MPa at 1200 °C and 1400 °C, respectively. Both stress–strain diagrams show a steady decrease in strength after yield points. No fracture was observed at 1000 °C, 1200 °C, and 1400 °C.

The Table 3 and Table 4 summarize the results of compressive tests. Comparing the performance at room temperature, HfMoTaTiZr alloy has the best yield strength 1600 MPa; HfMoNbTaTi has the highest 27% fracture strain; comprehensively, HfMoNbTaTi has the best mechanical properties (yield strength 1369 MPa and 27% fracture strain). The fracture strain increases from 4% for the HfMoTaTiZr alloy to 12% for HfMoNbTaTiZr. The presence of Ta can increase the yield strength but decrease the toughness. Ti seems to have no significant influence on strength, but it has a negative effect on toughness. Zr increases the strength; however, it strongly deteriorates the toughness at room temperature, because the fracture strain decreases from 27% for the HfMoNbTaTi alloy to 12% for HfMoNbTaTiZr. The presence of Mo, which has the highest shear modulus, increases the yield strength at room temperature from 929 MPa to 1512 MPa significantly. Nevertheless, the toughness decreases tremendously, fracture strain declines from > 50% to 12%. However, the presence of Nb decreases the yield strength only slightly from room temperature to 1000 °C and increases the yield strength by 38% at 1200 °C, but largely improves the room temperature fracture strain. This indicates that more Nb could be added for higher ductility.

The melting point of the elements in the alloy affects the strength performance at elevated temperature. For instance, at 1200 °C, HfMoTaTiZr alloy and HfMoNbTiZr alloy which have lower melting-point elements (Ta, Mo, Nb) have less strength; HfMoNbTaZr alloy and HfMoNbTaTi alloy which have higher melting-point elements (Ti, Zr) have better strength. Moreover, all the alloys with Mo present have much better strength than HfNbTaTiZr does. Therefore, Mo makes a significant contribution to strength at elevated temperature.

**Figure 4:**
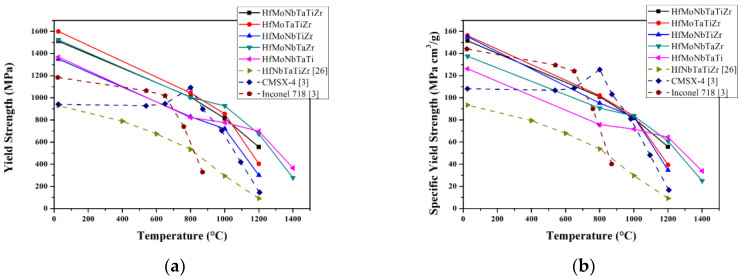
(**a**) Elevated temperature yield strength and (**b**) elevated temperature specific yield strength versus temperature between Hf-Mo-Nb-Ta-Ti-Zr alloy series, CMSC-4, and Inconel 718 [3]. The elevated temperature yield strength of HfNbTaTiZr is from Reference [26].

The elevated temperature yield strength versus temperature of Hf-Mo-Nb-Ta-Ti-Zr alloys is shown in Figure 4a. Except at 800 °C, the strength of the above mentioned Hf-Mo-Nb-Ta-Ti-Zr alloys is better than the commercial nickel base superalloys, CMSX-4 and Inconel 718. Additionally, Hf-Mo-Nb-Ta-Ti-Zr alloys also have better resistance to softening at elevated temperature. Figure 4b is the specific strength of Hf-Mo-Nb-Ta-Ti-Zr alloys, HfNbTaTiZr alloy, CMSX-4, and Inconel 718 at different temperatures. Below 900 °C, CMSX-4 and Inconel 718 perform better; at 1000 °C, Hf-Mo-Nb-Ta-Ti-Zr alloys, except for HfNbTaTiZr, are better than CMSX-4 and Inconel 718. At temperatures above 1200 °C, HfMoNbTaTi alloy has the highest specific strength.

Comprehensively speaking, Hf-Mo-Nb-Ta-Ti-Zr alloys have a potential application at elevated temperature.

## 4. Discussion

### 4.1. Phase Formation Rule

The solid solution phase formation rules were checked with the microstructure and crystal structure of the alloys in this study. First are the criterion based on thermodynamic parameters and atomic size parameter [33,34]. The thermodynamic parameters are mixing entropy *∆S_mix_*, mixing enthalpy *∆H_mix_*, and *Ω*, respectively:

(1) $$∆S_{\mathrm{mix}}=-\sum c_{i}{\ln c}_{i}$$

(2) $$∆H_{\mathrm{mix}}=\sum 4∆H_{\mathrm{ij}}c_{i}c_{j},\;i\;\neq \;j$$

(3) $$\Omega \;=\;\frac{T_{m}∆S_{\mathrm{mix}}}{∆H_{\mathrm{mix}}}$$

where *R* is the gas constant, *c_i_* is the atomic percentage of the element *i*, *c_j_* is the atomic percentage of the element *j*, ∆*H_ij_* is the enthalpy of the binary liquid state of elements *i* and *j* at an equiatomic composition from the Miedema’s model [35,36], and *T_m_* is the melting point of the alloy defined by rule of mixing:

(4) $$T_{m}\;=\;\sum c_{i}T_{m,i}$$

where *T_m,i_* is the melting point of the element *i*. The atomic size parameter is atomic size difference *δ*:

(5) $$\delta =\sqrt{\sum c_{i}{\left(1-\frac{r_{i}}{\bar{r}}\right)}^{2}}$$

where *r_i_* is the atomic radius of element *i*. $\bar{r}$ is the average radius of the alloy defined by rule of mixing.

(6) $$\bar{r}=\sum c_{i}r_{i}$$

The second criterion determining crystal type is related to electronic parameters, valence electron concentration *VEC* [37], and the third criterion determining Laves phase is related to Allen electronegativity difference Δ*χ_Allen_* [38]:

(7) $$\mathrm{VEC}=\sum c_{i}{\mathrm{VEC}}_{i}$$

(8) $$∆\chi_{\mathrm{Allen}}=\sqrt{c_{i}{\left(1-\frac{\chi_{i}^{\mathrm{Allen}}}{\bar{\chi }}\right)}^{2}}$$

where *VEC_i_* is the valence electron concentration of the element *i* [39], $\chi_{i}^{\mathrm{Allen}}$ is the electronegativity of the element *i* from Allen et al. [40], and $\bar{\chi }$ is the average electronegativity of the alloy defined by rule of mixing:

(9) $$\bar{\chi }=\sum c_{i}\chi_{i}^{\mathrm{Allen}}$$

The ranges in the three criterions for predicting the phases and crystal structure of HEAs are (1) Disorder solid solution phase forms when Ω >1.1 and *δ* < 6.6% [34]; (2) face-centered cubic (FCC) is stable when *VEC* > 8, and BCC is stable when *VEC* < 6.87 [37]; and (3) Laves phase forms when ∆*χ_Allen_* > 7% and *δ* > 5% in HEAs [41]. All the criterions mentioned above are established through statistical approach, so there is still some error especially in the boundary condition. The properties of pure elements Hf, Mo, Nb, Ta, Ti, and Zr are listed in Table 5. The BCC atomic radii of Hf, Ti, and Zr are Goldschmidt radii since all the alloys are a BCC structure. All the parameters of six alloys are calculated and listed in Table 6. From Table 5, one can observe Hf and Zr possess the largest atomic radius and smallest electronegativity, and Mo possesses the smallest atomic radius and biggest electronegativity. Furthermore, from the experiment results, all the alloys at the as-cast state form a single BCC disorder solid solution phase and no Laves phase is observed. This indicates that the formation of a single solid solution phase is consistent with criteria (1) and (2) but not consistent with criterion (3). Base on criterion (3), Laves phase might form in all the alloys except HfNbTaTiZr which is at the margin. It is necessary to check the criterion for Laves phase formation because Mo_2_Hf or Mo_2_Zr might form according to the Hf-Mo or Mo-Zr binary phase diagram. The result shows that criterion 3 is not fulfilled in the present alloy series. The minimum value 7% seems to be lower. One can observe that the Ω parameter values of these alloys are much higher than 1.1 and the VEC values are significantly lower than 6.87. This demonstrates that the high entropy effect is significant in enhancing the formation of a solid solution when mixing enthalpy and strain energy is small.

### 4.2. Solution Hardening Mechanism

The solid solution strengthening effect is calculated to examine the yield strength of the alloys in this study. From the experiment results, all the alloys possess a single phase of BCC disorder solid solution. It is valuable to use the yield strength of the alloys to check the solution strengthening mechanism. The solution strengthening mechanism of HEAs was proposed by Senkov et al. and then modified by Yao et al. [25,42]. The solution strengthening value ∆*σ_i_* contributed by element *i* is:

∆*σ_i_* = *AGf_i_*^4/3^*c_i_*^2/3^ (10)

where *A* is a material-dependent dimensionless constant of the order of 0.04, *G* is the shear modulus of the alloy, and *f_i_* is the mismatch parameter of element *i* related to shear modulus and atomic size:

(11) $$f_{i}\;=\;\sqrt{\delta_{G,i}{}^{2}{+\;\alpha }^{2}\delta_{r,i}{}^{2}}$$

where *δ_G,i_* and *δ_r,i_* are the modulus mismatch parameter and atomic radius mismatch parameter, respectively as Equations (12) and (13). The value of *α* depends on the type of dislocation. For mixed dislocation, the value is designated to be nine.

(12) $$\delta_{G,i}\;=\;\frac{9}{8}\sum^{}c_{j}\delta_{G,\mathrm{ij}}$$

(13) $$\delta_{r,i}\;=\;\frac{9}{8}\sum^{}c_{j}\delta_{r,\mathrm{ij}}$$

where *δ_G,ij_* and *δ_a,ij_* are the differences between elements *i* and *j* in shear modulus and atomic radius, respectively as Equations (14) and (15). Nine is the number of atoms in the *i*-centered cluster in the BCC lattice, eight is the number of atoms neighboring with the center atom *i*.

(14) $$\delta_{G,\mathrm{ij}}\;=\;\frac{{2(G}_{i}-G_{j})}{{(G}_{i}{\;+\;G}_{j})}$$

(15) $$\delta_{r,\mathrm{ij}}\;=\;\frac{{2(r}_{i}-r_{j})}{{(r}_{i}\;+\;r_{j})}$$

where *G_i_* and *G_j_* are the shear modulus of element *i* and *j*, respectively, and *r_j_* is the atomic radius of element *j*. Eventually, the solution strengthening ∆*σ* contributed by all the alloying elements is obtained by summation of ∆*σ_i_*. The calculated yield stress *σ_c_* is the summation of the yield stress, *σ_m_*, by rule of mixing and ∆*σ*.

(16) $$∆\sigma \;=\;(\sum^{}{\left(∆\sigma_{i}\right)}^{3/2}{)}^{2/3}$$

*σ_c_* = *σ_m_* + ∆*σ* (17)

As the shear modulus of the HfMoNbTaTiZr alloy system is still lacking, we reasonably use the rule of mixing to calculate it since the modulus relates to the interatomic potential energy well:

(18) $${\;G}_{m}\;=\;\sum^{}c_{i}G_{i}$$

**Figure 5:**
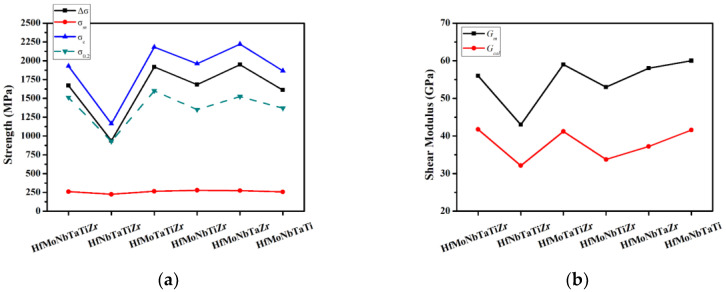
The trend of Hf-Mo-Nb-Ta-Ti-Zr alloy series: (**a**) ∆*σ*, *σ_m_*, *σ_c_*, and *σ*_0.2_, and (**b**) *G_m_* and *G_cal_*.

The calculated results are listed in Table 7 and compared in Figure 5. One can observe that the *σ_m_* of all the alloys is small and almost the same. In addition, the trends of ∆*σ* and *σ_c_* are consistent with *σ_0.2_*. This means that high yield strength of this alloy series all comes from solution strengthening effect despite there being some deviation, about 30%, between calculated values and experimental values. It is interesting to note that Mo, with the smallest atomic radius and the largest shear modulus, interacts frequently with other elements, thus, the HfNbTaTiZr alloy possesses the smallest yield stress without the addition of Mo. Ti, having the average atomic radius and the average shear modulus, interacts slightly with other elements, and, thus, HfMoNbTaZr alloy possess the largest ∆*σ* without the addition of Ti. As for the deviation between *σ_c_* and *σ_0.2_,* it might be due to the overestimated shear modulus. Young’s modulus of HfNbTaTiZr is 81 GPa reported by Juan et al. [43], and, thus, the shear modulus can be calculated to be 31 GPa. This value is obviously smaller than the average shear modulus 43 GPa of HfNbTaTiZr. This implies that all the average shear moduli might be overestimated. If we calculated the shear modulus from the experimental *σ_0.2_*, by assumption that *σ_c_* equals to *σ_0.2_*, the result is shown in the *G_cal_* column of Table 7. From the figure, the trend of calculated shear modulus *G_cal_* is consistent with the trend of average shear modulus although significantly smaller than G*_m_* by ~30%. One can observe that the calculated shear modulus of HfNbTaTiZr 32 GPa is in a good agreement with the literature [43]. This indicates that severe lattice distortion in HEAs has a strong solution hardening effect, but Young’s modulus was effectively lower. This is reasonable and could be related to its effect on lattice constant [11]. In the alloy series, NiCo, NiCoFe, NiCoFeCr, and NiCoFeCrMn, the lattice constant of real crystal structure has more deviation from that predicted by Vegard’s law. The increased lattice constants as compared with ideal average lattice constant indicates that lattice distortion has the effect to expand the lattice. Thus, the interatomic bonding strength is effectively lower and the shear modulus is simultaneously lower. However, in the present alloy series, the calculated lattice constants based on Vegard’s law are larger than the experimental lattice constants measured from X-ray diffraction patterns as listed in Table 2, especially with the addition of Mo. This is because Mo has a strong interaction with other elements to reduce the bond length according to ∆*H_ij_* in Table 5. Al has the same effect and is reported in Reference [27]. However, there needs to be more research in the future to confirm the reasons for the reduced shear modulus. 

## 5. Conclusions

The equiatomic HfMoNbTaTiZr alloy was chosen to analyze the effect of each constituent elemental by the subtraction method and, thus, HfMoNbTaTiZr, HfNbTaTiZr, HfMoTaTiZr, HfMoNbTiZr, HfMoNbTaZr, and HfMoNbTaTi were studied. Among these alloys, HfMoNbTaTi has the best mechanical performance, that is, 27% compressive strain at room temperature and yield strength 367 MPa at 1400 °C. HfMoNbTaTi has great potential for elevated-temperature applications. As the alloy system does not contain very expensive elements such as Re and Ru, it is cost competitive for high-temperature applications like Nb-Hf-Ti alloys in space vehicles. Further modification of composition and/or anti-oxidation coatings are still required for high-temperature applications in the air.

According to the experiment results, the effects of Mo, Nb, Ta, Ti, and Zr on mechanical properties of equiatomic Hf-Mo-Nb-Ta-Ti-Zr alloys were described. For higher room-temperature strength, one should add an element which interacts frequently with the alloy, such as Mo. For higher elevated-temperature strength, one should add the elements which possess high melting points, such as Mo, Nb, or Ta. One should add more Nb for higher ductility. With Ti or Zr addition, the elevated-temperature strength and the density decreases. All these elemental effects could also be applied to all other RHEAs systems, but more research is required to confirm this premise.

The solid solution phase formation rule and the solid solution strengthening effect of RHEAs have been discussed. The high entropy effect of the present alloys is significant in enhancing the formation of a solid solution. The shear modulus of RHEAs is smaller than that predicted from mixture rule by about 30%. This reduction is attributable to severe lattice distortion. 

## Figures and Tables

**Table 1:** Results of scanning electron microscope-energy dispersive spectrometry (SEM-EDS) analysis (at. %). Nominal composition means the designed composition. DR means the dendritic region. ID means the interdendritic region.

	Element	Hf	Mo	Nb	Ta	Ti	Zr
HfMoNbTaTiZr	Nominal	16.7	16.7	16.7	16.7	16.7	16.7
	DR	14.3	18.4	19.5	24.4	12.4	10.8
	ID	21.1	13.6	12.3	9.9	18.3	24.7
HfNbTaTiZr	Nominal	20.0	-	20.0	20.0	20.0	20.0
	DR	18.5	-	22.4	27.4	18.2	13.5
	ID	22.6	-	17.5	12.8	20.2	26.9
HfMoTaTiZr	Nominal	20.0	20.0	-	20.0	20.0	20.0
	DR	20.6	21.4	-	23.9	18.3	15.7
	ID	24.4	16.2	-	11.0	21.2	27.1
HfMoNbTiZr	Nominal	20.0	20.0	20.0	-	20.0	20.0
	Overall	20.8	20.6	19.7	-	19.2	19.7
HfMoNbTaZr	Nominal	20.0	20.0	20.0	20.0	-	20.0
	DR	18.5	20.8	21.7	24.5	-	14.5
	ID	27.0	15.6	13.5	9.9	-	34.0
HfMoNbTaTi	Nominal	20.0	20.0	20.0	20.0	20.0	-
	DR	15.5	22.7	19.5	25.7	16.6	-
	ID	30.7	16.9	17.6	10.7	24.1	-

**Table 2:** The lattice constants (Å) of the Hf-Mo-Nb-Ta-Ti-Zr alloy series. Cal. means the value calculated from Vegard’s Law. Exp. means the value calculated by Nelson–Riley extrapolation function based on X-ray diffraction pattern.

	HfMoNb TaTiZr	HfNbTa TiZr	HfMoTa TiZr	HfMoNb TiZr	HfMoNb TaZr	HfMoNb TaTi
Cal.	3.361	3.404	3.373	3.373	3.378	3.317
Exp.	3.345	3.400	3.364	3.369	3.347	3.305

**Table 3:** The room temperature compressive yield strength and fracture strain of the Hf-Mo-Nb-Ta-Ti-Zr alloy series.

	HfMoNb TaTiZr	HfNbTa TiZr [25]	HfMoTa TiZr	HfMoNb TiZr	HfMoNb TaZr	HfMoNb TaTi
Yield strength (MPa)	1512	929	1600	1351	1524	1369
Fracture strain (%)	12	> 50	4	20	16	27

**Table 4:** The elevated temperature compressive yield strength (MPa) of the Hf-Mo-Nb-Ta-Ti-Zr alloy series.

Temperature (°C)	HfMoNb TaTiZr	HfNbTa TiZr [25]	HfMoTa TiZr	HfMoNb TiZr	HfMoNb TaZr	HfMoNb TaTi
800	1007	535	1045	829	1005	822
1000	814	295	855	721	927	778
1200	556	92	404	301	694	699
1400	N. A.	N. A.	N. A.	N. A.	278	367

**Table 5:** Various data of the properties of Hf, Mo, Nb, Ta, Ti, and Zr. HCP means hexagonal close-packing.

∆*H_ij_* (kJ/mol)	Hf	Mo	Nb	Ta	Ti	Zr
Hf	-	−4	4	3	0	0
Mo	−4	-	−6	−5	−4	−6
Nb	4	−6	-	0	2	4
Ta	3	−5	0	-	1	3
Ti	0	−4	2	1	-	0
Zr	0	−6	4	3	0	-
*r_i_* (nm)	0.159 (HCP) 0.155 (BCC)	0.136	0.143	0.143	0.147 (HCP) 0.142 (BCC)	0.162 (HCP) 0.157 (BCC)
*T_m,i_* (K)	2506	2896	2750	3290	1941	2128
$\chi_{i}^{\mathrm{Allen}}$	1.16	1.47	1.41	1.34	1.38	1.32
*VEC_i_*	4	6	5	5	4	4
*G* (GPa)	30	120	38	69	44	33

**Table 6:** The values of thermodynamics, atomic size, and electronic parameters of the Hf-Mo-Nb-Ta-Ti-Zr alloy serious.

	HfMoNb TaTiZr	HfNbTa TiZr	HfMoTa TiZr	HfMoNb TiZr	HfMoNb TaZr	HfMoNb TaTi
∆*H_mix_* (kJ)	−0.9	2.7	−1.9	−1.6	−1.1	−1.4
∆*S_mix_* (J)	14.9	13.4	13.4	13.4	13.4	13.4
*T_m_* (K)	2585.2	2523.0	2552.2	2444.2	2714.0	2676.6
*Ω*	43.3	12.4	17.8	20.4	32.4	24.9
*δ*	6.3%	5.5%	6.7%	6.7%	6.9%	5.4%
VEC	4.7	4.4	4.6	4.6	4.8	4.8
Δ*χ_Allen_*	7.2%	6.6%	7.6%	7.8%	7.8%	7.8%

**Table 7:** Comparisons of G*_m_*, ∆*σ*, *σ_m_*, *σ_c_*, *σ*_0.2_, and G*_cal_* of the present alloy series.

	*G_m_* (GPa)	∆*σ* (MPa)	*σ_m_* (MPa)	*σ_c_* (MPa)	*σ*_0*.2*_ (MPa)	*G_cal_* (GPa)
HfMoNbTaTiZr	55	1669	260	1929	1512	41
HfNbTaTiZr	43	938	225	1163	929 [26]	32
HfMoTaTiZr	60	1918	264	2182	1600	41
HfMoNbTiZr	53	1683	278	1961	1351	33
HfMoNbTaZr	58	1948	273	2221	1524	37
HfMoNbTaTi	60	1610	256	1866	1369	41
